# Oral Cholera Vaccine Coverage during an Outbreak and Humanitarian Crisis, Iraq, 2015

**DOI:** 10.3201/eid2301.160881

**Published:** 2017-01

**Authors:** Eugene Lam, Wasan Al-Tamimi, Steven Paul Russell, Muhammad Obaid-ul Islam Butt, Curtis Blanton, Altaf Sadrudin Musani, Kashmira Date

**Affiliations:** Centers for Disease Control and Prevention, Atlanta, Georgia, USA (E. Lam, S.P. Russell, C. Blanton, K. Date);; World Health Organization, Baghdad, Iraq (W. Al-Tamimi, M.O.I. Butt, A.S. Musani)

**Keywords:** vaccines, oral cholera vaccine, cholera, refugees, internally displaced persons, refugee camps, internally displaced persons camps, internally displaced persons collective centers, humanitarian emergency, humanitarian crisis, immunization, vaccination, vaccine coverage, outbreaks, civil conflicts, enteric infections, bacteria, Iraq

## Abstract

During November–December 2015, as part of the 2015 cholera outbreak response in Iraq, the Iraqi Ministry of Health targeted ≈255,000 displaced persons >1 year of age with 2 doses of oral cholera vaccine (OCV). All persons who received vaccines were living in selected refugee camps, internally displaced persons camps, and collective centers. We conducted a multistage cluster survey to obtain OCV coverage estimates in 10 governorates that were targeted during the campaign. In total, 1,226 household and 5,007 individual interviews were conducted. Overall, 2-dose OCV coverage in the targeted camps was 87% (95% CI 85%–89%). Two-dose OCV coverage in the 3 northern governorates (91%; 95% CI 87%–94%) was higher than that in the 7 southern and central governorates (80%; 95% CI 77%–82%). The experience in Iraq demonstrates that OCV campaigns can be successfully implemented as part of a comprehensive response to cholera outbreaks among high-risk populations in conflict settings.

As of 2015, ≈3.2 million internally displaced persons (IDPs) were dispersed throughout Iraq due to increased activity by an armed antigovernment group and subsequent counter-insurgency operations by the Iraq government and coalition forces, and Iraq was hosting >200,000 Syrian refugees due to protracted fighting in Syria between the government and several opposition groups (*1*). The risk of communicable disease epidemics in Iraq is heightened due to the large numbers of displaced populations residing in camps, informal settlements, or temporary placement sites (collective centers). These sites are usually overcrowded and have inadequate shelter arrangements and limited access to sanitation facilities, safe drinking water, safe food, and basic healthcare services. Such risk factors, coupled with austerity measures and the effect of those measures on health services, have contributed to transmission of cholera in Iraq.

On August 30, 2015, cholera was confirmed in Iraq’s southern governorate, Diwaniya, and on September 15, an outbreak was declared by the Iraq Ministry of Health (MoH); activation of the Cholera Control and Command Center followed the outbreak declaration. The outbreak continued to rapidly spread throughout the country, and by October 2015, a total of 1,656 laboratory-confirmed cases of *Vibrio cholerae* 01 Inaba had been reported from 15 of 18 governorates; 1,000 (60%) of these cases were reported in Babylon and Baghdad, which are in southern and central governorates.

Oral cholera vaccines (OCVs) are recommended by the World Health Organization (WHO) as a complementary strategy for comprehensive cholera prevention and control in addition to the primary intervention of safe water, sanitation, and hygiene (WaSH) measures. Three OCVs are currently prequalified by WHO: Dukoral, Shanchol, and Euvichol (*2*,*3*). In early 2013, a global OCV stockpile was established with initial support from several donors and endorsed for funding support through Gavi, the Vaccine Alliance (*4*). The stockpile, which is intended to provide rapid deployment of OCVs in emergency and outbreak situations, is managed by the International Coordinating Group that comprises 4 decision-making partners: the International Federation of Red Cross and Red Crescent Societies; Médecins Sans Frontières; United Nations Children’s Fund; and WHO, which also serves as the Secretariat (*5*,*6*).

When the 2015 cholera outbreak began in Iraq, the Iraq MoH and implementing partners immediately began planning a vaccination campaign using the bivalent OCV Shanchol (*7*–*9*) to complement WaSH and other cholera control measures. The 2-dose OCV campaign targeted ≈255,000 persons >1 year of age living in selected refugee camps, IDP camps, and collective centers because of increased vulnerability to cholera due to living conditions. This deployment of ≈510,000 OCV doses in Iraq was the largest to date from the global OCV stockpile for outbreak and humanitarian response. As part of the recommended monitoring and evaluation activities for these deployments, the MoH requested partners to conduct a vaccination coverage survey to evaluate vaccine uptake, OCV campaign awareness, reasons for vaccine acceptance or nonacceptance, and any adverse events reported after the campaign. We report results of the coverage survey and key lessons learned from the Iraq experience.

## Methods

### Study Setting

Because of the large numbers of IDPs and the limited supply of OCV, the vaccination campaign in Iraq was limited to IDP camps at full capacity or overcrowded and to all refugee camps and collective centers. The OCV campaign was conducted during October 31–November 5, 2015 (round 1), and December 7–9, 2015 (round 2). Campaign dates were chosen beyond the 2-week minimum interval between OCV doses to accommodate a polio vaccination campaign that was conducted between the 2 OCV campaign rounds. Vaccination teams were trained by WHO and MoH staff and composed of at least 1 vaccinator, recorder, and crowd controller. Experiences from the polio vaccination teams and infrastructure supported the implementation of this campaign during a public health emergency. Vaccination strategy included a combination of fixed sites (i.e., large health centers) and mobile teams for door-to-door vaccine delivery. The vaccine cold chain was maintained, and vaccines were transported using a sufficient number of vaccine carriers and ice packs for a door-to-door strategy. The coverage survey was conducted during December 14–16, 2015, immediately after the second round of the campaign, by WHO, the US Centers for Disease Control and Prevention, and the Iraqi Red Crescent Society.

### Study Design

We designed a stratified multistage cluster survey to obtain representative OCV coverage estimates among selected camps in Iraq’s governorates that were targeted during the 2015 campaign. The sampling universe was stratified first by governorate and then by refugee camp, IDP camp, or collective center within a governorate. Within each household, all persons in each of 3 designated age groups (1–4, 5–14, and >15 years of age) were interviewed.

We performed sample size calculations based on an estimated 2-dose coverage of 75%, an intraclass correlation of 0.2, an average household size of 6, and a nonresponse rate of 5%. Based on these assumptions, we estimated a design effect of 2 due to household clustering. To achieve 8% precision in the group of 1- to 4-year-old children for the northern and southern/central regions, we estimated that ≈120 households per governorate would need to be sampled and allocated the sample equally to each governorate. We expected to yield a coverage estimate with a precision of 4.7% for each governorate.

Within each governorate, we proportionally allocated our sample based on the estimated population size of each refugee camp, IDP camp, or collective center. For logistical reasons, we excluded camps that had a population of <500 persons. A total of 35 camps and collective centers were eligible for sampling, but in the governorates of Anbar and Baghdad Karkh, we selected only the 2 largest camps due to security concerns, logistical challenges, and access issues in the southern and central regions. All eligible camps in the northern region were selected. Overall, we selected 27 refugee camps, IDP camps, and collective centers in 10 governorates for this survey; 3 governorates were in the northern region (Dahuk, Erbil, Sulaymaniya), and 7 were in the southern and central regions (Najaf, Baghdad Karkh, Kerbala, Salah Addin, Anbar, Wasit, Babil).

Within a selected camp, the allocated numbers of households were systematically sampled using a predetermined skip interval, which we calculated as the estimated number of households in the camp divided by the proportionally allocated sample size. Survey teams used a start, selected randomly between the first household at the corner of the camp and the nth household, based on the predetermined sampling interval. Once the interview at the first household was completed, the interviewers moved on to the next household, based on the sampling interval. Selected households that were excluded because of ineligibility (if consent was not given or if no one was present at the household after 3 attempted visits) were counted toward the sample size per camp; that is, selected households were not replaced for nonresponse or refusal reasons. In camps or collective centers where population size was larger than estimated, the survey team continued to sample households using the predetermined skip interval.

In each selected household, all eligible persons were interviewed; for younger children, information was collected from parents or the primary caregiver. If any of the household members were absent during the first visit, teams attempted to revisit the household at least twice at a later time. Respondents were categorized by 3 age groups: 1–4 years, 5–14 years, and >15 years to match administrative recording via tally sheets for the campaign and to align with data from previous campaigns in other settings (*9*–*12*). All data were collected using the Survey123 application (Esri, Redlands, CA, USA) installed in electronic tablets for real-time data entry and global positioning system (GPS) tracking of survey teams. Electronic data entry and GPS tracking of survey teams were used to remotely monitor the spatial pattern of selected households and data quality during this humanitarian crisis in a complex security environment.

### Analytic Methods

We used survey procedures in SAS version 9.3 (SAS Institute, Cary, NC, USA) for data analysis. Data were weighted to ensure that each individual in the sampling frame had an equal probability of selection and to adjust for potential nonresponse bias. We had to account for 3 different stages of weighting: 1) probability of selection by taking the inverse of the sampling rate, 2) household nonresponse rate by calculating the inverse of the governorate-wide household response rate, and 3) individual nonresponse by calculating the inverse of the response rate within a household. The Iraq MoH approved this survey as a program evaluation activity.

## Results

### Response Rate and Household Characteristics

We selected 1,240 households in the targeted camps and collective centers to survey; 99% (1,226 households; 5,007 persons) participated. The governorate of Dahuk had the lowest household response rate (93%), followed by Erbil and Baghdad Karkh (99% each); the remaining 7 governorates all had a 100% response rate. Among 5,007 individual-level survey respondents in the 10 governorates, 51% were female, 10% were 1–4 years of age, 22% were 5–14 years of age, and 69% were >15 years of age (Table 1). The median number of residents per household was 4 (interquartile range 3–5). The governorate of Anbar did not report household-level questions and therefore was excluded from the household-level analysis. Overall, 12% of households reported using an unimproved primary water source, 36% reported using an unimproved secondary water source, and 4% reported having an unimproved sanitation facility (Table 1). Among all households, 22% reported sharing sanitation facilities with >4 other households, and 4% reported not having soap for handwashing.

**Table 1:** Individual and household characteristics for oral cholera vaccination survey respondents in refugee camps, internally displaced persons camps, and collective centers targeted for vaccination, Iraq, 2015

Characteristic	No.	Weighted estimate, % (95% CI)
Individual level, n = 5,007		
Sex		
Male	2,487	49 (47–51)
Female	2,500	51 (49–53)
Age, years		
1–4	650	10 (9–11)
5–14	1,235	21 (19–24)
>15	3,117	69 (66–71)
Household level, n = 1,226		
Water sources*		
Unimproved primary water source	458	12 (10–14)
Unimproved secondary water source	666	36 (31–42)
Sanitation facilities		
Unimproved†	85	4 (2–6)
Shared with >4 other households	366	22 (18–26)
Lacked soap for handwashing	86	4 (2–7)

*Unimproved water sources include unprotected well; unprotected spring water; river, stream, lake, irrigation, or canal water; bottled water; water truck; and water vendor. †Unimproved sanitation sources include pit latrines without cement slab; bucket toilet; hanging toilet or hanging latrine; and canal, open, bush, and field defecation.

### OCV Coverage

Among the 5,007 respondents from the 10 governorates, 87% reported 2-dose OCV coverage, and 7% reported 1-dose coverage (Table 2). Two-dose coverage was similar among male (86%) and female (88%) respondents and among age groups: 85% among children 1–4 years of age, 89% among children 5–14 years of age, and 87% among persons >15 years of age (Table 2). When vaccination coverage was stratified by sex and age group, the lowest 2-dose coverage was among boys 1–4 years of age (83%) and the highest was among girls 5–14 years of age (89%). OCV campaign vaccination cards were available for 79% of persons who reported being fully vaccinated; these cards indicated that 47% had received 2 doses, and 32% had received 1 dose. Among the respondents who reported receiving 2 doses, 27% had only 1 dose recorded on their vaccination cards. Among the respondents who reported receiving OCV, 90% reported receiving the vaccine at their residential structure, 6% at a health facility, 3% at school, and 1% at a market.

**Table 2:** Estimated oral cholera vaccine coverage, by vaccinee age, among persons in refugee camps, internally displaced persons camps, and collective centers targeted for vaccination, Iraq, 2015*

Vaccination group, no. doses	All vaccinees			Male vaccinees			Female vaccinees	
	No. vaccinated	Weighted estimate, % (95% CI)		No. vaccinated	Weighted estimate, % (95% CI)		No. vaccinated	Weighted estimate, % (95% CI)
Overall								
2 doses	3,523	87 (85–89)		1,735	86 (83–89)		1,777	88 (85–90)
1 dose	745	7 (6–9)		363	8 (6–11)		380	7 (5–9)
1–4 y of age								
2 doses	407	85 (81–88)		213	83 (77–88)		191	85 (81–89)
1 dose	89	5 (3–7)		50	6 (4–9)		38	4 (2–7)
5–14 y of age								
2 doses	931	89 (85–92)		487	88 (83–92)		442	89 (84–93)
1 dose	171	7 (5–11)		88	9 (5–14)		83	6 (3–11)
>15 y of age								
2 doses	2,183	87 (84–90)		1,034	86 (83–89)		1,143	88 (84–91)
1 dose	483	8 (6–10)		224	8 (6–12)		258	8 (6–10)
*Zero doses and incomplete data are not shown.								

Two-dose OCV coverage in the northern governorates (91%) was higher than that in the southern and central governorates (80%), and 1-dose coverage in the northern governorates (6%) was lower than that in the southern and central governorates (10%) (Table 3). Among the northern governorates, 2-dose OCV vaccination coverage ranged from 90% in Dahuk to 93% in Erbil and Sulaymaniya; however, greater variability was seen between the southern and central governorates, where 2-dose coverage ranged from 21% in Babil to 98% in Anbar (Figure; Table 3).

**Table 3:** Estimated oral cholera vaccine coverage, by region and governorate, among refugee camps, internally displaced persons camps, and collective centers targeted for vaccination, Iraq, 2015*

Region, governorates	Frequency	No. doses	Weighted estimate, % (95% CI)
Northern, N = 154,396	1,340	2	91 (87–94)
		1	6 (4–9)
Dahuk, n = 98,327	351	2	90 (84–94)
		1	7 (4–12)
Erbil, n = 34,675	476	2	93 (90–96)
		1	4 (2–7)
Sulaymaniya, n = 21,394	513	2	93 (89–96)
		1	6 (4–10)
Southern and central, N = 78,046	3,667	2	80 (77–82)
		1	10 (8–12)
Anbar, n = 41,070	539	2	98 (93–99)
		1	2 (1–6)
Wasit, n = 2,300	523	2	91 (85–95)
		1	6 (3–12)
Salah Addin, n = 6,000	528	2	81 (74–86)
		1	16 (11–22)
Najaf, n = 14,000	572	2	74 (67–81)
		1	10 (6–16)
Baghdad Karkh, n = 6,055	471	2	37 (29–44)
		1	28 (20–36)
Kerbala, n = 6,017	529	2	30 (22–38)
		1	35 (28–42)
Babil, n = 2,604	505	2	21 (15–27)
		1	31 (24–39)

**Figure:**
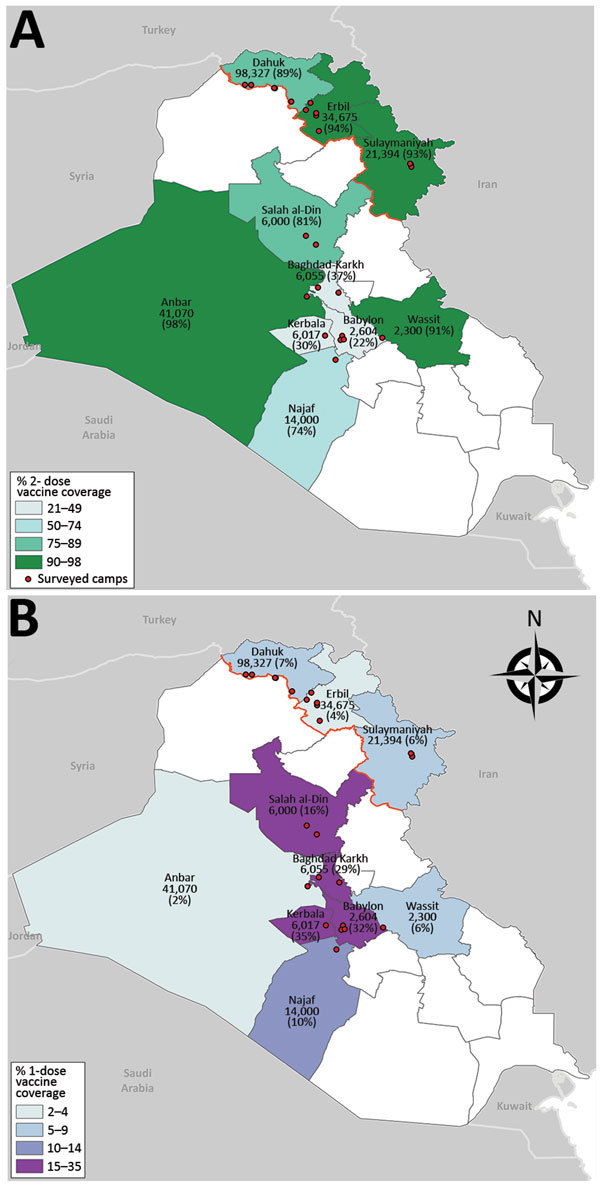
Location of camps and collective centers where persons were surveyed and vaccinated during a cholera outbreak and humanitarian crisis, Iraq, 2015. Numbers indicate targeted population; estimated 2-dose (A) and 1-dose (B) oral cholera vaccine coverages are shown in parentheses. White indicates governorates where surveys and vaccination were not conducted; black outlining indicates governorates; red line indicates border between the northern region and the southern and central regions of Iraq.

### Reasons for Not Being Vaccinated

The 2 most common reasons for not receiving vaccine during the first or second OCV vaccination round were being absent during the campaign (first round 35%, second round 39%) and teams not visiting the respondents’ residential structures (first round 30%, second round 36%) (Table 4). Other reasons for not being vaccinated during the first round were unavailability of vaccine (11%), lack of faith in the vaccine (4%), and being sick during the campaign (3%). The reasons for not being vaccinated during the second round were similar: unavailability of vaccine (2%), sick during the campaign (9%), and absence of the decision-maker at home at the time of the vaccinators’ visit (5%). In the 3 governorates with the lowest coverage (Baghdad Karkh, Kerbala, and Babil), 46% of respondents stated vaccination teams did not visit their residential structure, and 22% stated they were absent during the campaign.

**Table 4:** Five most common reasons for not receiving oral cholera vaccine among persons in refugee camps, internally displaced persons camps, and collective centers targeted for vaccination, Iraq, 2015

Reasons for non-vaccination	Frequency	Weighted estimate, % (95% CI)
First dose		
Was absent during campaign	175	35 (27–43)
Teams did not visit my house	284	30 (23–38)
Vaccines not available	99	11 (7–18)
No faith in vaccine	22	4 (2–9)
Was sick	24	3 (2–5)
Second dose		
Was absent during campaign	148	39 (28–51)
Teams did not visit my house	419	36 (26–46)
Vaccines not available	38	2 (1–3)
Was sick	22	9 (4–18)
Decision-maker not at home	2	5 (2–18)

### Adverse Events following Vaccination

Adverse events within 14 days of receiving either the first or second dose of OCV were reported by 21% of respondents. The most commonly reported adverse events were minor, primarily abdominal pain (9%), fever (5%), vomiting (3%), and diarrhea (2%). Only 1 person reported rash following vaccination. No severe adverse events were reported.

### OCV Campaign–Associated Messaging

Most vaccine recipients reported having received information about the OCV campaign through television (19%), neighbors or friends (13%), radio (12%), health staff (7%), or posters or banners posted before or during the campaign (7%) (Table 5). In addition, 55% of respondents reported receiving other cholera prevention messages, such as handwashing (33%), thoroughly cooking food (14%), boiling water (15%), and washing vegetables and fruits (13%).

**Table 5:** OCV campaign information sources and cholera-associated messages reported seen or heard by survey respondents in refugee camps, internally displaced persons camps, and collective centers targeted for vaccination, Iraq, 2015*

Information source and type	Frequency	Weighted estimate, % (95% CI)
Source for information about campaign		
Television	1,046	19 (15–24)
Neighbors or friends	599	13 (10–16)
Radio	315	12 (9–17)
MoH staff or vaccinators	579	7 (5–10)
Poster or banner	521	7 (5–9)
Schools	88	3 (2–6)
Community mobilizer	62	3 (2–6)
SMS text messages	57	3 (2–5)
Mosque announcements	62	1 (0.3–3)
The Internet	40	<1 (<0.1–0.2)
Received nonvaccine information during campaign	2,410	55 (49–61)
Nonvaccine information heard or seen during campaign		
Wash hands with soap and water	2,023	33 (28–39)
Cook food thoroughly	930	14 (11–18)
Wash vegetables and fruits	1,413	13 (10–16)
Boil water	794	15 (12–18)
Clean cooking utensils and vessels	635	6 (4–8)
Dispose of human waste properly	519	5 (4–7)
Drink and use water treated with chlorine products	334	4 (3–7)
Go to health center if I have cholera	312	4 (3–6)
Take ORS if I have cholera	163	3 (1–6)
Do not know or do not remember	35	<1 (0.1–0.4)
*MoH, Ministry of Health; OCV, oral cholera vaccine; ORS, oral rehydration solution; SMS, short message service.		

## Discussion

We describe the context of an OCV campaign in Iraq that was conducted during an acute humanitarian emergency and cholera outbreak and results from an OCV coverage survey in the vaccine-targeted areas. The primary objective of vaccination in an acute humanitarian emergency is to rapidly reduce disease risk to protect a population during periods of extreme vulnerability (*13*). The risk for cholera epidemics among displaced populations during a humanitarian crisis can be elevated, especially due to massive population movements and overcrowding. Limited access to clean water, adequate sanitation, and shelter are also risk factors associated with cholera epidemics. The World Health Assembly (WHA) and WHO have recommended OCV use in the context of a humanitarian emergency to reduce morbidity and mortality from cholera, where indicated. In 2011, because of worldwide increases in cholera incidence, the WHA adopted resolution WHA 64.15, which called for implementation of an integrated and comprehensive approach to cholera control, including rapid provision of safe water, adequate case management at health facilities, strengthened case detection through early-warning surveillance and laboratory confirmation, and cholera vaccination (*14*).

The use of the global OCV stockpile was a positive experience during the humanitarian crisis and outbreak response in Iraq. The rapidity of the OCV response activity is highlighted by the short time that passed between cholera detection and implementation of the OCV campaign. During ≈1 month, cholera was detected, a request for OCV was submitted to the International Coordinating Group, the decision to provide OCV was made, vaccine was deployed and arrived in country, and the first round of the OCV campaign was planned and implemented. Excellent collaboration and coordination was seen among partners, not only for campaign implementation but also for evaluation activities.

Administrative coverage data have several limitations for immunization programs in general, usually because of issues with population denominator estimates (*15*,*16*). Coverage surveys can help verify administrative data and provide helpful insights into the reasons for vaccine acceptance or nonacceptance and the effectiveness of social mobilization activities. Two-dose coverage among targeted camps in Iraq was high (87%) compared with OCV campaigns conducted in other conflict settings (*10*,*17*,*18*) and with campaigns conducted in more stable, conflict-free settings (*19*–*22*). 

Coverage among the OCV-targeted camps in Iraq’s northern governorates was high; national authorities thought this high coverage reflected the commitment and dedication of country staff and partners and the use of adaptive vaccination strategies during campaign implementation. Certain governorates were able to attain high coverage (e.g., 98% in Anbar) due to the strongly captive nature of the closed camps and collection centers, which restricted movement of the populations in or out. Compared with the northern governorates, the southern and central governorates had lower 2-dose coverage, especially in Baghdad Karkh, Kerbala, and Babil. Civil strife, heavy rains, and challenges in program management might have played a role. Although 2 OCV doses are recommended, a recent single-dose trial in Bangladesh showed promising results (*23*), and a modeling study showed that single-dose coverage may be especially useful for interrupting disease transmission in outbreak situations that present challenges to population access (*24*). Furthermore, vaccine thermostability data that support considerations for the use of controlled temperature chains for OCV may help eliminate stringent cold chain requirements, thereby simplifying vaccine delivery (*25*).

OCV campaign vaccination cards did not accurately portray 2-dose vaccination status for all respondents, even though the coverage survey was implemented immediately after the campaign. This finding suggests a need to remind vaccine recipients to bring back vaccination cards for the second round and to improve vaccination card recording training for vaccination teams. Previous OCV campaigns in Haiti that reported higher card-documented 2-dose coverage (51%–70%) emphasized the value of keeping vaccination cards for receiving the second vaccine dose (*21*). Where feasible, the use of serologic studies may also be helpful in validating reported coverage.

A unique feature of the coverage survey in this complex security environment during a humanitarian crisis was the use of electronic tablets for data collection and GPS tracking of survey teams, which enabled remote review of data quality, spatial tracking of teams, and immediate feedback for corrective actions. In addition to using fixed posts at health facilities, the Iraqi MoH adopted a door-to-door strategy for both rounds of the OCV campaigns. This strategy was based on the MoH’s familiarity with the use of this method in previous polio and measles campaigns. The door-to-door strategy explains the finding that most respondents (90%) were vaccinated at residential structures. Furthermore, strategies to include extended evening hours and additional sites at camp entrances and nearby markets helped capture male persons >15 years of age, a group that has experienced the lowest coverage rates in other campaign settings (*20*,*21*). However, given that one of the most frequent reasons for not being vaccinated was that vaccination teams did not visit residential structures, clearer messaging may be needed for the dual strategy of providing vaccine door-to-door and at fixed posts.

Less than 10% of respondents reported receiving information about the campaign from MoH staff or community mobilizers, which may have been because of the complex management structure of numerous partners and nongovernmental organizations within the targeted camps. It was encouraging to note that at least half of all survey participants reported receiving educational information on cholera other than OCV (e.g., WaSH measures) during the campaign. Advance communications to health clinics and nongovernmental organizations to strengthen social mobilization and messaging activities, especially WaSH and vaccine integration, will be helpful for comprehensive cholera prevention and control in the future.

The camps and collective centers selected for the OCV campaigns were targeted because they were overcrowded or at full capacity, and it was believed that such facilities were most at risk for cholera because of high population density. However, our survey results showed that >85% of respondents had access to improved primary water sources, improved sanitation facilities, and soap. Future campaigns may also consider inclusion of communities outside of camps, which may have worse WaSH conditions than their in-camp counterparts. Many communities were not targeted in this campaign because the supply of vaccine at the global level was limited. Efforts to increase the global OCV supply available for outbreaks and humanitarian emergencies will help expand access to more settings in need.

Our survey had limitations. First, survey enumerators from southern and central governorates were unable to attend a centralized training and therefore relied on training by local supervisors, which may have resulted in varied training. Second, the survey results by region and governorate should only represent the selected camps and collective centers surveyed. However, access to the camps and centers was severely limited during the campaign and monitoring activities because of safety issues; access was especially difficult in the 3 governorates with the lowest coverage, where vaccination teams had difficulty visiting residential structures. Hence, it is possible that the camps that were excluded from the survey due to access issues were also the ones that were missed during the campaign. We were not able to visit smaller camps in southern and central governorates because they were in areas of heavy conflict, and therefore the assessment is not representative of these areas.

The OCVs Shanchol and Euvichol are relatively new additions to cholera and acute humanitarian response activities, although there have been some prior examples of their use in emergency settings (*18*,*20*,*26*). Given the current vaccine supply limitations, monitoring and evaluation activities form an integral component of OCV stockpile use. These activities provide information to help determine the most appropriate use of vaccine; the factors associated with vaccine acceptance; and, where possible, the effect of vaccine use on disease transmission. Although it was determined that extensive evaluations would not be possible in Iraq, partners did agree that a coverage survey was feasible and would provide insights into campaign implementation, strategies, and acceptability and key lessons learned in this unique setting. Because an oral polio vaccine campaign was scheduled around the same time as the OCV campaign, the 2 OCV rounds were scheduled about a month apart. No data are currently available regarding co-administration of oral polio vaccine with OCV; such data are critically needed to optimize vaccine delivery in resource-limited settings without compromising the effectiveness of either vaccine. Nevertheless the use of polio eradication assets and activities to support public health emergencies contributed to the success in implementing the OCV campaign in Iraq. This experience of optimizing polio eradication assets, infrastructure, and experience in this survey was unique and proves the principle of the legacy of polio eradication efforts in action (*27*).

Vaccination is one of the most basic and critical health interventions for protecting vulnerable populations during emergencies. The Iraq experience has shown that OCV campaigns can be part of a comprehensive response to cholera outbreaks among populations at high-risk in conflict settings. OCV use in humanitarian emergencies should complement the foundational public health interventions (i.e., appropriate case management, enhanced environmental control, improved WaSH measures, and social mobilization) of all cholera prevention and control programs (*14*).
